# P-289. Short-Duration vs. Long-Duration of Therapy for Hospital-Acquired or Ventilator-Associated Pneumonia due to Multi-Drug Resistant *Pseudomonas aeruginosa*

**DOI:** 10.1093/ofid/ofae631.492

**Published:** 2025-01-29

**Authors:** Mohammed Al Musawa, Kaylee E Caniff, Chloe Judd, Michael Veve, Michael J Rybak

**Affiliations:** Wayne State University, Detroit, Michigan; Anti-Infective Research Lab, Eugene Applebaum College of Pharmacy and Health Sciences, Wayne State University, Royal Oak, Michigan; Wayne State University, Detroit, Michigan; Henry Ford Health, Detroit, Michigan; Eugene Applebaum College of Pharmacy and Health Sciences, Detroit, Michigan

## Abstract

**Background:**

The duration of therapy for hospital-acquired pneumonia (HAP) or ventilator-associated pneumonia (VAP) due to non-lactose fermenter Gram-negatives bacilli including *Pseudomonas aeruginosa* (PsA) remains a topic of debate. Our study aimed to compare the efficacy of short-duration (≤8 days) to long-duration ( >8 days) therapy for HAP/VAP due to multi-drug resistant (MDR) or difficult-to-treat (DTR) PsA infection.

**Methods:**

A two-center, retrospective cohort study was conducted from 5/2010 to 12/2022. We included all patients ≥18 years old with HAP or VAP due to MDR- or DTR-PsA in respiratory cultures who were treated for ≥72 hours. The primary outcome was a 30-day recurrence from the index culture, and the secondary outcomes were a 60-day recurrence, 30-day, and 60-day mortality from the index culture.

**Results:**

Overall, 203 patients (short duration, n=91 and long duration, n=112) were included. Sixty-nine percent were male, and 58.6% were of African American descent. The mean (SD) for age and APACHE II score were 58.5 (15.8) and 23.9 (7.9), respectively. The common comorbidities were diabetes (39.9%), cerebrovascular disease (28.6%), and chronic obstructive pulmonary disease (24.1%). The primary admission source was a nursing home (44.8%). VAP was diagnosed in 53.0% of patients, with DTR-PsA accounting for 58.1% of all infections. Polymicrobial infection was present in 20.1% of the cases with *Enterobactarales* species present in all cases. Ceftazidime-avibactam and ceftolozane-tazobactam were used for treatment in 33.0% and 47.8%, respectively; combination therapy was used for 11.8% of cases. The 30-day recurrence was significantly lower in the long-duration therapy compared with the short-duration (5.4% vs. 15.9%, p = 0.017). Similarly, there was a lower 60-day recurrence rate in the long-duration group vs. short-duration group (8.9% vs. 24.4%, p=0.026). There were no significant differences in 30-day and 60-day mortality rates observed.

**Conclusion:**

Prolonged duration ( >8 days) has resulted in lower recurrence rates of HAP/VAP caused by MDR/DTR PsA infection at 30 and 60 days. Although our study is insightful, a larger-scale study is necessary, and logistic regression should be conducted to determine the predictors of the significant differences in the primary outcome.

**Disclosures:**

**Kaylee E. Caniff, PharmD, BCIDP**, T2Biosystems: Honoraria **Michael J. Rybak, PharmD, PhD, MPH**, Abbvie, Melinta, Sionogi, Merck, T2Biosystems: Advisor/Consultant|Abbvie, Melinta, Sionogi, Merck, T2Biosystems: Grant/Research Support|Abbvie, Melinta, Sionogi, Merck, T2Biosystems: Speaker

